# Differential cell reaction upon Toll-like receptor 4 and 9 activation in human alveolar and lung interstitial macrophages

**DOI:** 10.1186/1465-9921-11-124

**Published:** 2010-09-15

**Authors:** Jessica Hoppstädter, Britta Diesel, Robert Zarbock, Tanja Breinig, Dominik Monz, Marcus Koch, Andreas Meyerhans, Ludwig Gortner, Claus-Michael Lehr, Hanno Huwer, Alexandra K Kiemer

**Affiliations:** 1 Pharmaceutical Biology, Saarland University, Saarbrücken, Germany; 2 Department of Virology, Saarland University Hospital, Homburg, Germany; 3 Department of Neonatology, Saarland University Hospital, Homburg, Germany; 4 Leibniz Institute for New Materials, Saarland University, Saarbrücken, Germany; 5 Infection Biology Group, ICREA and University Pompeu Fabra, Barcelona Biomedical Research Park, Barcelona, Spain; 6 Department of Biopharmaceutics and Pharmaceutical Technology, Saarland University, Saarbrücken, Germany; 7 Department of Cardiothoracic Surgery, Herzzentrum Saar, Völklingen, Germany

## Abstract

**Background:**

Investigations on pulmonary macrophages (MΦ) mostly focus on alveolar MΦ (AM) as a well-defined cell population. Characteristics of MΦ in the interstitium, referred to as lung interstitial MΦ (IM), are rather ill-defined. In this study we therefore aimed to elucidate differences between AM and IM obtained from human lung tissue.

**Methods:**

Human AM and IM were isolated from human non-tumor lung tissue from patients undergoing lung resection. Cell morphology was visualized using either light, electron or confocal microscopy. Phagocytic activity was analyzed by flow cytometry as well as confocal microscopy. Surface marker expression was measured by flow cytometry. Toll-like receptor (TLR) expression patterns as well as cytokine expression upon TLR4 or TLR9 stimulation were assessed by real time RT-PCR and cytokine protein production was measured using a fluorescent bead-based immunoassay.

**Results:**

IM were found to be smaller and morphologically more heterogeneous than AM, whereas phagocytic activity was similar in both cell types. HLA-DR expression was markedly higher in IM compared to AM. Although analysis of TLR expression profiles revealed no differences between the two cell populations, AM and IM clearly varied in cell reaction upon activation. Both MΦ populations were markedly activated by LPS as well as DNA isolated from attenuated mycobacterial strains (*M. bovis *H37Ra and BCG). Whereas AM expressed higher amounts of inflammatory cytokines upon activation, IM were more efficient in producing immunoregulatory cytokines, such as IL10, IL1ra, and IL6.

**Conclusion:**

AM appear to be more effective as a non-specific first line of defence against inhaled pathogens, whereas IM show a more pronounced regulatory function. These dissimilarities should be taken into consideration in future studies on the role of human lung MΦ in the inflammatory response.

## Introduction

Macrophages (MΦ) are cells of the body's defence system widely distributed in the peripheral and lymphoid tissues. They differentiate from monocytes, which represent leukocytes circulating in the blood. MΦ are phagocytic cells and act both in the innate as well as in the acquired immune system. MΦ express MHC-II molecules and therefore function as antigen-presenting cells. In addition, MΦ secrete numerous cytokines making them key factors in the modulation of immune functions. The production of pro-inflammatory cytokines by macrophages, such as TNF-α, induces a typical Th1, i.e. a pro-inflammatory immune response. On the other hand, macrophages can also induce a Th2 response by secreting anti-inflammatory mediators, such as IL10 [1].

Alveolar macrophages (AM) located in lung alveoli play a central role in pulmonary innate immunity as the first line of defence against inhaled particles and pathogens. Besides their function in the defence against infectious diseases they are known to play a role in inflammatory airway diseases, such as chronic obstructive pulmonary disease (COPD) [2] and to regulate immune responses in allergic disease [3].

In contrast to alveolar macrophages as a rather well-defined macrophage population, which are commonly obtained by bronchoalveolar lavage (BAL), little is known about another potential macrophage-like cell population in human lungs referred to as lung interstitial macrophages (IM).

Studies using primary rat or mouse macrophages suggest that AM are more effective than IM in producing cytokines involved in an antimicrobial defence whereas IM express higher levels of MHC-II molecules and have a more pronounced accessory function [4,5]. The relevance of these observations is not described in the literature. One of the very few studies investigating functional differences between human AM and IM describes a phagocytic activity of AM compared to IM [6]. Moreover, a higher production of matrix metalloproteinases in IM compared to AM [7] has been reported, indicating that IM might play a more pronounced role in tissue remodelling.

Lung dendritic cells have recently gained marked scientific interest. This cell type resides in small numbers in the lung interstitial tissue in close proximity to both the large airways and the alveoli and is specialized for antigen presentation and accessory function [4,8,9]. A study using mouse models only recently revealed that IM are able to inhibit maturation and migration of lung dendritic cells [5]. This makes IM the cell type responsible for the suppression of allergic reactions towards harmless antigens. The relevance of these findings for humans, however, need to be confirmed.

Over the last several years, Toll-like receptors (TLRs) have emerged as important transducers of the innate immune response. TLRs act as a first line of host immunity against various pathogens. Presently, ten human TLRs are known, which recognize pathogen-associated molecular patterns including bacterial cell wall components such as lipoproteins (TLR1/2 or TLR1/6 dimers) or lipopolysaccharide (LPS, TLR4), bacterial flagellin (TLR5), viral RNA (TLR3, 7 and 8) as well as bacterial DNA (TLR9) [10].

In order to investigate the role of AM and IM in the pathogenesis of human lung disease, aim of the present study was to characterize respective cell populations isolated from human lung tissue. Since Toll-like receptors represent key mediators of infectious [11] as well as non-infectious lung disease [12] a special focus was laid on potential differences in AM and IM with respect to activation *via *TLR4 and TLR9.

## Methods

### Materials

FITC-labelled anti-CD14 (61/D3) and FITC-IgG1 were obtained from eBioscience (San Diego, CA, USA), PE-labelled anti-HLA-DR (AB3), PE-labelled anti-CD68 (KP1), FITC-labelled anti-CD1a (NA1/34) as well as PE-IgG2a κ, FITC-IgG2a κ and PE-IgG1 κ isotype controls were purchased from Dako (Carpinteria, CA, USA). PE-labelled anti-CD83 (HB15e), PE-labelled CD90 (5E10), and PE-IgG1 κ were from BD Biosciences (San Jose, CA, USA). Other chemicals were obtained from Sigma-Aldrich (St. Louis, MO, USA) or Roth (Karlsruhe, Germany) if not marked otherwise.

### Bacterial culture

Mycobacteria (*M. bovis *BCG, wild-type *M. bovis*, H37Rv, H37Ra) were grown in Middlebrook 7H9 broth containing 10% ADC, 0.2% glycerol and 0.05% Tween 80 (7H9-ADCT) or on Middlebrook 7H10 agar containing OADC (Becton Dickinson, Franklin Lake, NJ, USA), 0.5% glycerol and antifungal cycloheximide (100 μg/ml) (Sigma-Aldrich, St. Louis, MO, USA). Antibiotics included hygromycin (50 μg/ml) and kanamycin (25 μg/ml).

### Cell culture

#### Alveolar macrophages

Alveolar macrophages were isolated from human non-tumor lung tissue, which was obtained from patients undergoing lung resection. The use of human material for isolation of primary cells was reviewed and approved by the local Ethics Committees (State Medical Board of Registration, Saarland, Germany). Isolation was performed referring to a protocol for the recovery of type II pneumocytes previously described by Elbert et al. [13]. After visible bronchi were removed, the lung tissue was sliced into pieces of about and washed at least three times with BSS (balanced salt solution; 137 mM NaCl, 5 mM KCl, 0.7 mM Na_2_HPO_4_, 10 mM HEPES, 5.5 mM glucose, pH 7.4). The washing buffer was collected and cells were obtained by centrifugation (15 min, 350 × *g*). Remaining erythrocytes were lysed by incubation with hypotonic buffer (155 mM NH_4_Cl, 10 mM KHCO_3_, 1 mM Na_2_EDTA) and the cell suspension was washed with PBS (137 mM NaCl, 2.7 mM KCl, 10.1 mM Na_2_HPO_4_, 1.8 mM KH_2_PO_4_, pH 7.4) three times. Subsequently, cells were resuspended in MΦ medium (RPMI 1640, 5% FCS, 100 U/ml penicillin G, 100 μg/ml streptomycin, 2 mM glutamine), seeded at a density of 0.5-1 × 10^6 ^cells/well in a 12- or 6-well plate and incubated at 37°C and 5% CO_2 _for 2 h. Adherent cells were washed at least 5 times with PBS and cultivated with medium for 3-4 days. Medium was changed every two days.

#### Lung interstitial macrophages

After recovering alveolar macrophages, lung tissue was chopped into pieces of 0.6 mm thickness using a McIlwain tissue chopper. To remove remaining alveolar macrophages and blood cells, the tissue was washed with BSS over a 100 μm cell strainer until the filtrate appeared to be clear. The tissue was then digested using a combination of 150 mg trypsin type I (T-8003, Sigma-Aldrich, Carpinteria, CA, USA) and 0.641 mg elastase (LS022795, CellSystems, Remagen, Germany) in 30 ml BSSB for 40 min at 37°C in a shaking water bath. After partial digestion, the tissue was brought to DMEM/F12 medium (PAA, Pasching, Austria) containing 25% FCS (PAA, Pasching, Austria) and 350 U/ml DNase I (D5025, Sigma-Aldrich, St. Louis, MO, USA). Remaining undigested lung tissue in the solution was disrupted by repeatedly pipetting the cell suspension slowly up and down. After filtration through gauze and a 40 μm cell strainer, cells were incubated with a 1:1 mixture of DMEM/F12 medium and SAGM (Cambrex, East Rutherford, NJ, USA), containing 5% FCS and 350 U/ml DNase I in Petri dishes in an incubator at 37°C and 5% CO_2 _for 90 min in order to let macrophages attach to the plastic surface. Afterwards, non-adherent cells were removed by washing with PBS. As surface receptor expression might be influenced by different isolation procedures, cells were cultured with MΦ medium for 3-4 days to restore receptors as shown previously for tissue macrophages isolated by enzyme perfusion [14]. Medium was changed every other day.

#### Isolation of monocytes and cultivation of DCs

Monocytes were isolated from healthy adult blood donors (Blood Donation Center, Saarbrücken, Germany) as described by Schütz et al. [15]. Briefly, peripheral blood mononuclear cells (PBMCs) were isolated from buffy coats using Ficoll-Paque (Amersham Biosciences, Piscataway, NJ, USA). The cell layer containing mononuclear cells was washed in PBS, erythrocytes lysed, and washed again twice with PBS. Subsequently, cells were allowed to adhere to culture flasks for 2 h at 37°C. Non-adherent cells were removed by washing, and the adherent monocytes were harvested. To generate immature DCs (iDC), monocytes were cultured for 5 d in the presence of GM-CSF (800 U/ml, Berlex Bioscience Inc., Richmond, CA, USA) and IL-4 (20 U/ml, Strathmann Biotec, Hamburg, Germany) with one-quarter of the medium being replaced by fresh cytokine-containing medium on day 2 post-isolation. Mature dendritic cells (mDC) were generated by adding 100 ng/ml LPS (Sigma-Aldrich, St. Louis, MO, USA) to iDC cultures for an additional 48 h.

### Pappenheim staining

Air-dried MΦ preparations were stained using May-Grünwald solution (Roth, Karlsruhe, Germany) for 5 min, followed by addition of the same volume of distilled water and incubation for another 5 min, after which the staining solution was removed. Subsequently, preparations were incubated with Giemsa solution (1:20; Roth, Karlsruhe, Germany) for 15 min, washed with distilled water and visualized using light microscopy.

### RNA isolation and reverse transcription

Total RNA was extracted using either RNeasy mini or micro kit columns (Qiagen, Hilden, Germany). DNA was digested during the RNA isolation procedure using the RNase-Free DNase 1 treatment kit (Qiagen, Hilden, Germany). 500 ng of RNA were denatured at 65°C for 5 min, placed on ice, and then reverse transcribed in a total volume of 20 μl using the High-Capacity cDNA Reverse Transcription Kit (Applied Biosystems, Foster City, CA, USA) according to the manufacturer's instructions.

### Real-time quantitative PCR

The iCycler iQ5 (Bio-Rad, Richmond, CA, USA) was used for real-time quantitative PCR. Primers and dual-labelled probes were obtained from Eurofins MWG Operon (Ebersberg, Germany). Sequences are given in table 1 and 2. Standards, from 10 to 0.0001 attomoles of the PCR product cloned into pGEMTeasy (Promega, Heidelberg, Germany), were run alongside the samples to generate a standard curve. All samples and standards were analyzed in triplicate. The PCR reaction mixture consisted of 10 × PCR buffer (GenScript, Piscataway, NJ, USA), either 2 or 8 mM dNTPs, 3-9 mM Mg^2+^, 500 nM sense and antisense primers, either 2.5 or 1.5 pmol of the respective dual-labelled probe, and 2.5 U of *Taq *DNA Polymerase (GenScript, Piscataway, NJ, USA) in a total volume of 25 μl. The reaction conditions were 95°C for 8 min followed by 40 cycles of 15 s at 95°C, 15 s at a reaction dependent temperature varying from 57-60°C, and 15 s at 72°C. The starting amount of cDNA in each sample was calculated using the iCycler iQ5 software package (Bio-Rad, Richmond, CA, USA).

**Table 1:** Primer sequences as used for real time RT-PCR

	primer sense, 5'→3'	primer antisense, 5'→3'
**TLR1**	AGCAAAGAAATAGATTACACATCA	TTACCTACATCATACACTCACAAT
**TLR2**	GCAAGCTGCGGAAGATAATG	CGCAGCTCTCAGATTTACCC
**TLR3**	GAATGTTTAAATCTCACTGC	AAGTGCTACTTGCAATTTAT
**TLR4**	ATGAAATGAGTTGCAGCAGA	AGCCATCGTTGTCTCCCTAA
**TLR5**	GTACAGAAACAGCAGTATTTGAG	TCTGTTGAGAGAGTTTATGAAGAA
**TLR6**	TTTACTTGGATGATGATGAATAGT	AGTTCCCCAGATGAAACATT
**TLR7**	CCATACTTCTGGCAGTGTCT	ACTAGGCAGTTGTGTTTTGC
**TLR8**	AAGAGCTCCATCCTCCAGTG	CCGTGAATCATTTTCAGTCAA
**TLR9**	GGGACAACCACCACTTCTAT	TGAGGTGAGTGTGGAGGT
**TLR10**	CAACGATAGGCGTAAATGTG	GAACCTCGAGACTCTTCATTT
**TNF-α**	CTCCACCCATGTGCTCCTCA	CTCTGGCAGGGGCTCTTGAT
**IL10**	CAACAGAAGCTTCCATTCCA	AGCAGT TAGGAAGCCCCAAG
**IL6**	AATAATAATGGAAAGTGGCTATGC	AATGCCATTTATTGGTATAAAAAC
**β-Actin**	TGCGTGACATTAAGGAGA AG	GTCAGGCAGCTCGTAGCTCT

**Table 2:** Probe sequences as used for real time RT-PCR

	probe, 5' FAM →3' BHQ1
**TLR1**	ATTCCTCCTGTTGATATTGCTGCTTTTG
**TLR2**	ATGGACGAGGCTCAGCGGGAAG
**TLR3**	TTCAGAAAGAACGGATAGGTGCCTT
**TLR4**	AAGTGATGTTTGATGGACCTCTGAATCT
**TLR5**	AGGATCTCCAGGATGTTGGCTG
**TLR6**	GTCGTAAGTAACTGTCZGGAGGTGC
**TLR7**	ATAGTCAGGTGTTCAAGGAAACGGTCTA
**TLR8**	TGACAACCCGAAGGCAGAAGGCT
**TLR9**	ACTTCTGCCAGGGACCCACGG
**TLR10**	ATTAGCCACCAGAGAAATGTATGAACTG
**TNF-α**	CACCATCAGCCGCATCGCCGTCTC
**IL10**	AGCCTGACCACGCTTTCTAGCTGTTGAG
**IL6**	TCCTTTGTTTCAGAGCCAGATCATTTCT
**β-Actin**	CACGGCTGCTTCCAGCTCCTC

### Isolation of mycobacterial DNA

Before DNA isolation, bacteria were centrifuged and boiled for 10 min. DNA was isolated according to a previously published method [16]. Isolation was performed under sterile conditions in order to avoid bacterial contamination from the surrounding area. Additional precipitation and washing steps were included to assure purity of the DNA [17]. We checked all DNA preparations with a commercially available LAL assay (sensitivity 0.03 EU/ml; Cambrex, East Rutherford, NJ, USA) in order to exclude LPS contaminations. Moreover, absence of contaminants was confirmed for all DNA preparations by DNase treatment as well as methylation as described previously [16].

### Flow cytometry

MΦ were detached from the plates in TEN buffer (40 mM Tris, 1 mM EDTA, 150 mM NaCl) before staining. For extracellular staining of CD83 and CD1a, MΦ or DC were washed with PBS, resuspended in FACS buffer I (PBS containing 2.5% (v/v) bovine calf serum and 0.05% (w/v) NaN_3_) and then divided into aliquots, each containing up to 1 × 10^6 ^cells. Each aliquot was incubated with a specific or isotype control antibody for 30 min on ice. The cells were washed in FACSwash and resuspended in 1% (w/v) cold paraformaldehyde in PBS, pH 7.6. HLA-DR and CD14 staining were performed similarly, except that FACS buffer II (PBS containing 0.05% (w/v) NaN_3 _and 0.5% (w/v) BSA for HLA-DR) or III (PBS with 1% (w/v) NaN_3 _and 0.5% (w/v) BSA for CD14) were used instead of FACS buffer I. Intracellular staining of CD68 was done using the IntraStain Reagents (Dako, Carpinteria, CA, USA) according to the manufacturer's instructions. The stained cells were examined on a FACSCalibur, and results were analysed using the CellQuest software (BD Biosciences, San Jose, CA, USA). Results are reported as relative mean fluorescence intensity (MFI; mean fluorescence intensity of specifically stained cells related to mean fluorescence intensity of isotype control).

### Phagocytosis Assay

#### Sample preparation

To visualize the uptake of microspheres by MΦ, cells were incubated with 1.75 μm latex beads (Fluoresbrite Carboxylated YG microspheres; Polysciences, Warrington, PA, USA) at a 100:1 bead/cell ratio for 4 h in medium containing 5% FCS. To block fluoresphere uptake, cytochalasin D (10 μg/ml, Sigma-Aldrich, St. Louis, MO, USA) was added 1 h prior to addition of latex beads. Alternatively, MΦ were pretreated by incubation for 1 h at 4°C and further incubated with fluorespheres at the same temperature as the pretreatment. After the incubation of MΦ with latex beads, cells were washed 4-5 times with ice cold PBS to remove remaining fluorospheres, and detatched from plates using trypsin/EDTA buffer (PAA, Pasching, Austria). After washing with PBS, cells were assessed for fluorosphere uptake by flow cytometry or confocal laser scanning microscopy.

#### Flow cytometry assessment of fluorosphere uptake

Upon washing MΦ, cells were resuspended in ice-cold PBS, examined on a FACSCalibur and results were analysed using the CellQuest software (BD Biosciences, San Jose, CA, USA).

#### Confocal laser scanning microscopy

AM and IM were fixed for 10 min in PBS supplemented with paraformaldehyde 3.7%, permeabilized for 10 min with 0.25% Triton X-100, subsequently blocked for 30 minutes with BSA 1% in PBS and stained with rhodamin-phalloidine (Sigma-Aldrich, St. Louis, MO, USA) and TOTO-3 iodide (Invitrogen, Carlsbad, CA, USA). Images were captured using a LSM 510 Meta (Carl Zeiss, Oberkochen, Germany).

### Cytokine measurement

AM and IM were seeded at a density of 1 × 10^5 ^cells per well in 96 well plates. On day 4 post seeding, cells were incubated in a total volume of 100 μl medium in the presence or absence of LPS (100 ng/ml) for 6 h. The supernatants were collected and stored at -80°C until use in the multiplex cytokine assay. For cytokine measurement, a Milliplex MAP Human Cytokine Kit (Millipore, Billerica, MA, USA) was used, containing the following cytokines: IL1β, IL1ra, IL6, IL10, IL12p40, IL-12p70 and IFNγ. The immunoassay procedure was performed using a serial dilution of the 10,000 pg/ml human cytokine standard according to the manufacturer's instructions and the plate was read at the Luminex 200 System (Luminex, Austin, TX, USA). Total cellular protein concentrations were determined by Pierce BCA protein assay (Fisher Scientific, Nidderau, Germany) using a Sunrise absorbance reader (Tecan, Grödig, Austria) according to the manufacturer's instructions.

### Electron Microscopy

AM and IM were fixed with 0.12 M PBS supplemented with 1% (w/V) paraformaldehyde and 1% (w/V) glutardialdehyde. Wet samples were washed with distilled water before mounting on a Peltier stage cooling the sample down to 276 K. After purging the vacuum chamber in wet conditions samples were carefully dried to P = 500 Pa and measured under a tilting angle of 45° and an accelerating voltage of E = 5 kV with a Quanta 400 ESEM FEG (FEI, Hillsboro, OR, USA).

### Statistics

Data analysis and statistics were performed using Origin software (OriginPro 7.5G; OriginLabs, Northampton, MA, USA). All data are displayed as mean values ± SEM. Statistical differences were estimated by independent two-sample t-test. Differences were considered statistically significant when P values were less than 0.05.

## Results

### Cell number and appearence

The AM and IM fractions obtained from 30 - 50 g of lung tissue each contained 2-20 × 10^6 ^cells, with the number of IM being equal to or exceeding the number of AM. The overall viability of cells obtained by washing or enzyme digestion of lung tissue was > 90% as determined by trypan blue staining.

Both AM and IM preparations almost exclusively contained highly auto-fluorescent cells compared to low fluorescent cells like DC, as observed by flow cytometry and fluorescence microscopy (data not shown).

**Figure 1:**
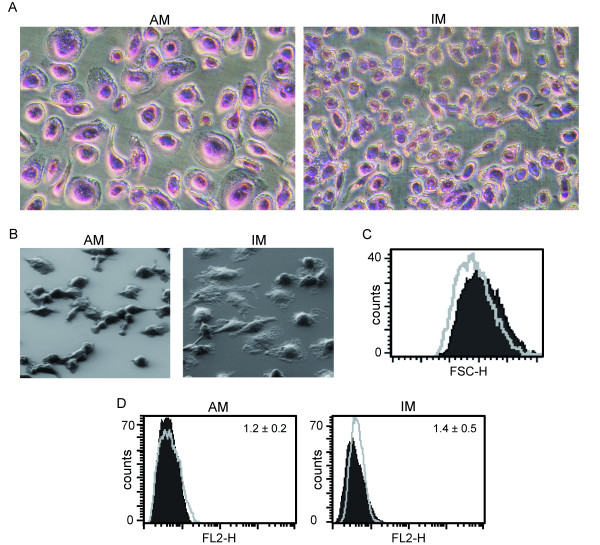
**Morphology and CD90 staining**. MΦ visualization by Pappenheim staining (A) and electron microscopy (B). Images are representative for cell preparations from at least two different donors. C: Comparison of MΦ sizes by forward scatter as measured by flow cytometry. Light grey line: IM; filled/dark grey: AM. D: CD90 staining of AM and IM. Filled/dark grey: isotype control; light grey line: antibody staining. MFI values are given within graphs. Data show one representative out of three independent experiments with cells obtained from different donors.

AM populations consisted mostly of large, round cells heterogeneous in size whereas IM appeared to be smaller but more heterogeneous in shape compared to AM as observed by light and electron microscopy (figure 1A, B). FACS analysis assessing FSC confirmed the smaller size of IM (figure 1C).

Phenotypic differences could be seen directly after isolation and persisted for at least 5 days. As tissue macrophages isolated by enzyme perfusion have been shown previously to require several days to recover surface receptor functionality [14], cells were cultured 3-4 days before use for further experiments.

Since the presence of fibroblasts can alter phagocyte functions [18,19] we determined a potential contamination with this cell type. However, neither AM nor IM exhibited a significant contamination with fibroblasts as shown by immunostaining of CD90. The surface marker is highly expressed in fibroblasts [20,21], as we confirmed for the human fibroblast cell lines MRC-5 and HSF-1 (data not shown). In contrast, CD90 is expressed only to a very low extent in macrophages, as was shown in the literature [20,21] and confirmed by ourselves in human differentiated THP-1 macrophages (data not shown). CD90 staining of AM and IM preparations revealed that mean percentages of CD90 positive cells were very low (0.9 ± 0.5% in AM *vs. *1.3 ± 0.5% in IM) and did not significantly differ between the two cell types (figure 1D).

### Expression of intracellular and surface markers

**Figure 2:**
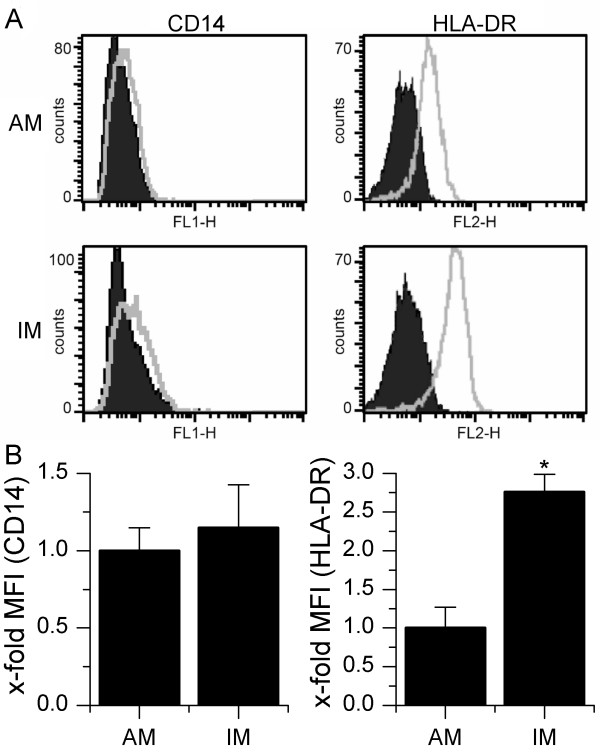
**CD14 and HLA-DR expression**. AM and IM were stained and analyzed by flow cytometry. A: Data show one representative out of four independent experiments. Filled/dark grey: isotype control; light grey line: antibody staining. B: Comparison of AM and IM concerning CD14 and HLA-DR expression. Data are expressed as MFI related to AM values. Data show means ± SEM of four independent experiments with cells derived from four different donors. *P < 0.05 compared to AM values.

**Figure 3:**
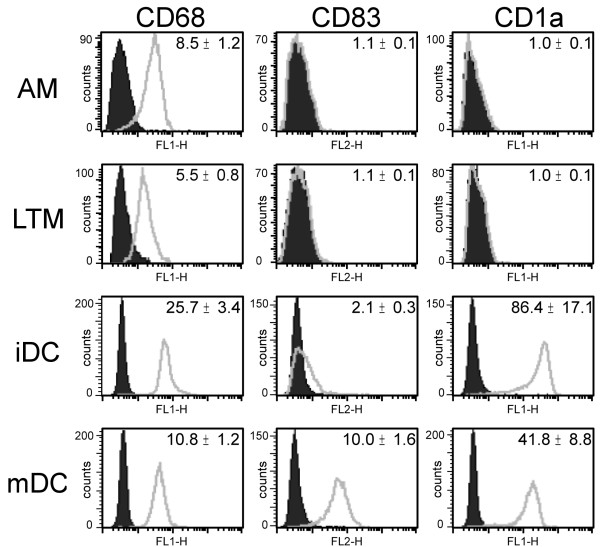
**Expression of CD68, CD83 and CD1a**. AM and IM as well as *in vitro *differentiated iDC and mDC were stained and analyzed by flow cytometry. Filled/dark grey: isotype control; light grey line: antibody staining. MFI values are given within graphs. Data show one representative out of three independent experiments with cells originating from different donors.

In order to define potential phenotypic differences between AM and IM, we analyzed their expression of the cell-surface molecules CD14 and human leukocyte-associated antigen-DR (HLA-DR). Moreover, the expression of surface markers CD83 and CD1a as well as intracellular CD68 in both populations was compared to *in vitro *differentiated iDC and mDC. Among the cell-surface molecules studied, only the expression of HLA-DR displayed significant differences between IM and AM, whereas CD14 expression was low or not detectable in both cell types (figure 2A, B). With respect to donor dependent differences in absolute MFI values, HLA-DR-expression in IM was almost 3-fold higher than in AM. CD68, often used as a specific marker for MΦ [5,22,23], was highly expressed in both AM and IM, but could also be found in iDC as well as mDC. The dendritic cell markers CD1a and CD83 were not detectable in both AM and IM (figure 3). These data suggest that IM share many phenotypic characteristics with AM, whereas no similarities to dendritic cells were observed.

### Phagocytosis

**Figure 4:**
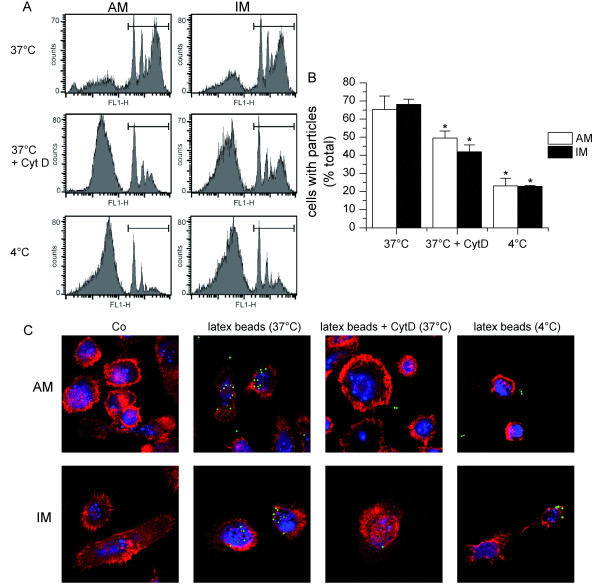
**Phagocytic Activity**. AM and IM were cultured with fluorescent FITC-labeled microspheres for 4 h at 37°C. As a control experiment, cells were pretreated with cytochalasin D (10 μg/ml, CytD) for 1 h. Alternatively, cells were preincubated at 4°C for 1 h and incubated with microspheres for 4 h at 4°C afterwards. Experiments were performed with cells derived from at least three different donors. A, C: representative results are shown. A: Fluoresphere-associated fluorescence (marked with black bars) was detected in AM and IM using flow cytometry. B: Average of percentage of MΦ positive for fluorosphere-associated fluorescence. Data represent mean ± SEM. *P < 0.05 as compared to cells left untreated at 37°C. C: Particle uptake in AM and IM was visualized by CLSM. F-actin was stained with rhodamin-phalloidine (red), nuclei with TOTO-3 iodide (blue). Latex beads are shown in green. Co: untreated cells.

The internalization of fluorescent latex beads by MΦ was quantified by flow cytometry. After incubation with fluorescent particles for 4 h, about two thirds of both MΦ populations had internalized fluorespheres. Particle uptake was significantly lowered by the pretreatment of the cells with cytochalasin D or incubation with fluorospheres at 4°C, but it was not abrogated completely (figure 4A and 4B). This might be due to particle attachment to the cell surface, which can not be distinguished from particle internalization by flow cytometry. Therefore, fluorosphere uptake was visualized by confocal laser scanning microscopy. Upon incubation with the fluorescent particles for 4 h, most MΦ had internalized several fluorospheres. As most of the particles were found to be internalized and not attached to the surface, quenching was supposed not to be necessary for flow cytometry analysis. Pretreatment with cytochalasin D or incubation at 4°C for 1 h prior to particle addition blocked particle uptake completely (figure 4C). Pre-treatment of MΦ with DMSO, the solvent used for cytochalasin D, did not affect particle uptake (data not shown).

### Toll-like receptor expression

**Figure 5:**
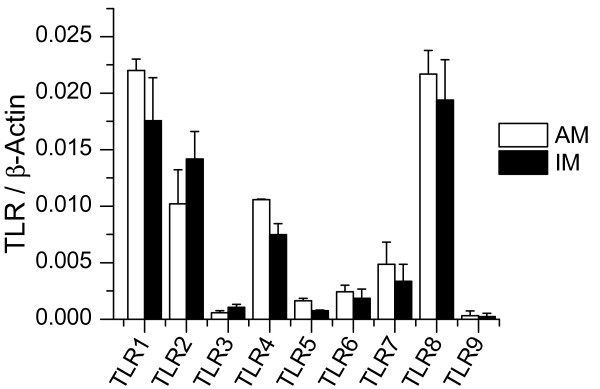
**Toll-like receptor expression**. RNA was isolated from AM and IM and real-time RT PCR analysis for TLR1-10 was performed. Data were normalized to β-Actin values. Data show means ± SEM of independent experiments performed with cells from 3 to 4 different donors.

To investigate the expression of TLR1-10, we performed real time RT-PCR with samples from untreated AM and IM. TLR mRNA expression levels were not significantly different in AM and IM (figure 5). Among the TLRs recognizing bacterial patterns, TLR1, 2 and 4 were expressed strongest, whereas TLR8 as a sensor of viral infections showed highest expression of the RNA-responsive receptors.

### Cell reaction upon TLR4/9 stimulation

**Figure 6:**
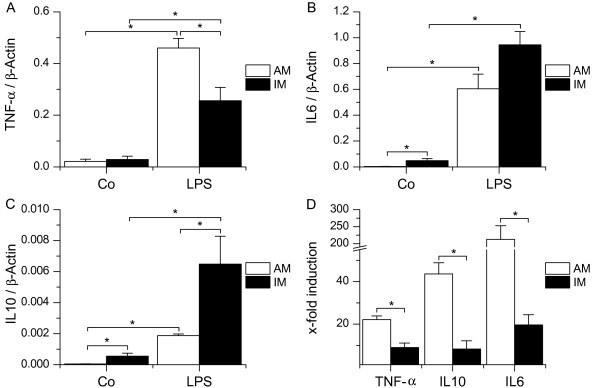
**Activation of AM and IM by LPS**. AM or IM were left untreated (Co) or treated with LPS (100 ng/ml) for 4 h, followed by RNA isolation and real-time PCR analysis for TNF-α (A), IL6 (B) or IL10 (C). Data are normalized to β-Actin values. D: Comparison of x-fold cytokine mRNA inductions. Data show means ± SEM of four independent experiments with cells derived from different donors. *P < 0.05.

As most comparative data for AM and IM focuses on TLR4 activation, we treated respective cell populations with LPS and then determined induction of cytokine mRNA. Though we observed an increase in TNF-α, IL10 and IL6 mRNA in both cell types, the extent of TNF-α induction observed in IM was weak compared to the increase of cytokine induction in AM. IM expressed both more IL6 and IL10 mRNA upon TLR4 activation than AM (figure 6). Interestingly, AM and IM differed also largely in basal IL10 and IL6 mRNA levels with IL10 expression in IM exceeding IL10 expression in AM 9.7-fold (± 2.4) and IL6 expression in IM being 16.9-fold (± 3.8) higher compared to AM (figure 6). These high basal expression levels of IL6 and IL10 in IM are also the reason why x-fold cytokine mRNA inductions upon TLR4 activation compared to respective untreated controls were higher in AM for all cytokine mRNAs investigated (figure 6D).

**Figure 7:**
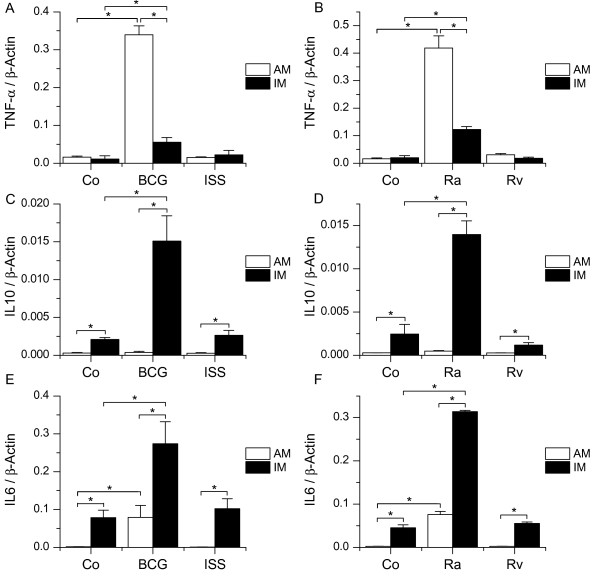
**Activation of AM and IM by TLR9 ligands**. AM or LTM were left untreated (Co) or incubated with TLR9 ligands, followed by RNA isolation and real-time PCR analysis for TNF-α (A, B), IL10 (C, D) or IL6 (E, F). A, C, E: Cells were treated either with immunostimulatory sequences (ISS 1018 phosphorothioate-modified oligonucleotide, 20 μg/ml) or genomic DNA from *M. bovis *BCG (20 μg/ml) for 2 h. B, D, F: DNA isolated from virulent *M. tuberculosis *(H37Rv) or from the attenuated H37Ra strain (20 μg/ml) was added to AM or LTM for 2 h. Data show means ± SEM of four experiments with cells derived from two different donors. *P < 0.05.

AM have only recently been shown to be highly activated by BCG DNA as TLR9 ligand despite low TLR9 expression levels [16]. Due to this interesting fact, we decided to also test responsiveness of IM towards TLR9 ligands. Cells were treated with different stimuli including a CpG-containing oligonucleotide (phosphorothioate-modified immunostimulatory sequence ISS 1018, 5'-TGACTGTGAACGTTCGAGATGA-3') and genomic DNA isolated from the attenuated *M. bovis *BCG strain. As reported previously for *in vitro *differentiated MΦ [16], TNF-α induction by ISS was weak or absent in both cell types. Treatment with BCG DNA resulted in a markedly stronger TNF-α induction in AM, but an only moderate response in IM (figure 7A). Interestingly, AM completely lacked IL10 induction upon stimulation with BCG DNA, whereas IM showed a distinct IL10 induction upon TLR9 activation (figure 7C). IL6 was induced in both cell types (figure 7E). The extent of IL10 as well as IL6 induction by ISS was minimal in both AM and IM.

Next, we examined cell reaction upon treatment with DNA from virulent (H37Rv) or attenuated (H37Ra) *mycobacteria. *Both AM and IM treated with DNA from virulent bacteria (H37Rv) showed a minimal induction of TNF-α compared to cells treated with DNA from non-virulent *Mycobacteria*(H37Ra, figure 7B; BCG, figure 7A). The lack of IL10 and IL6 induction by H37Rv DNA confirmed its low activatory potential (figure 7D, F). Observations for H37Ra DNA complied with the findings for BCG-DNA for both AM and IM, i.e. high TNF-α induction and absence of IL10 induction in AM contrasting a distinct IL10 response in IM.

**Figure 8:**
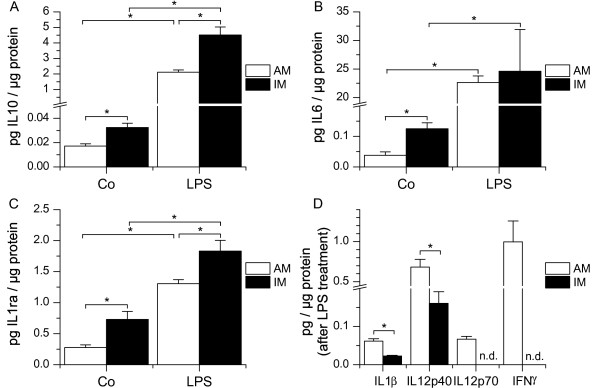
**LPS-induced cytokine secretion**. AM or IM were left untreated (Co) or treated with LPS (100 ng/ml) for 6 h. Supernatants were removed and used for measurement of cytokine protein production. Data are normalized to total cellular protein values. Data show means ± SEM of 2-4 independent experiments performed in triplicate with cells derived from different donors. *P < 0.05.

Taken together, these data obtained on mRNA level suggested that the activation profiles of AM and IM upon TLR4 and TLR9 stimulation are markedly different, indicating that both cell types clearly differ in functional properties. We therefore extended cytokine mRNA profiling of IL10 and IL6 to protein quantification using a fluorescent bead-based immunoassay and additionally determined the cytokine levels of IL1 receptor antagonist (IL1ra), IL1β, IL12p40, IL12p70, and interferon (IFN)-γ at baseline and after LPS activation in AM and IM. These data revealed that AM and IM constitutively produced IL10, IL6, and IL1ra. Most remarkably, the baseline production of these anti-inflammatory and regulatory cytokines was markedly higher in IM than in AM. In detail, IL10 secretion was 1.9-fold (± 0.2), IL6 secretion 3.3-fold (± 0.4), and IL1ra production 2.5-fold (± 0.4) higher in IM compared to AM (figure 8A-C). Upon LPS treatment, IM still produced significantly more IL10 as well as IL1ra than AM. In contrast, production of the proinflammatory cytokines IL1β and IL12p40 following LPS activation was significantly higher in AM compared to IM. IFNγ and IL12p70 were actually only secreted by AM, but not by IM, upon LPS challenge (figure 8D).

The production of higher amounts of inflammatory cytokines in AM compared to IM did not induce cell death as determined by MTT assay (data not shown).

## Discussion

### Isolation procedure

Human IM are less accessible than AM, which is why IM have in the past mostly been characterized using animal models [4,24]. Our approach for MΦ isolation from human lung interstitial was based on a previously described method for isolation of epithelial cells [13] and allows parallel isolation of AM, IM and epithelial cells. The digestion procedure that we used slightly differed from those previously described for isolation of human IM [6,7].

We are aware that donor specifics such as medication or smoking behaviour might alter MΦ functions. In fact, smoking has been shown previously to increase basal levels of TNF-α, IL1 or IL8 in human AM [25] and to cause changes in morphology and surface molecule expression in rat AM [4]. Nevertheless, our results reveal many similarities of AM from lung tissue compared to AM from bronchoalveolar lavage described in the literature, as detailed below.

### Morphology

Human as well as rat AM were previously described as large, mature cells, which closely resemble other tissue macrophages [6,24]. In contrast, IM of human, rat or hamster origin were shown to be smaller than AM, more uniform in size and to generally resemble more closely peripheral blood monocytes [5,24,26,27]. Our findings were similar to those described in the literature, which indicates that we were able to successfully separate MΦ populations.

### CD14 and HLA-DR expression

Studies using primary rat AM and IM suggest that AM and IM do not differ in CD14 expression [4]. We were able to show that CD14 is marginally expressed in human AM as well as IM. Low CD14 expression was reported previously for human AM obtained from bronchoalveolar lavage [28,29]. CD14 expression by human IM is not described in the literature, but our results resemble findings reported for other tissue macrophage populations [28].

Several studies using rat, mouse as well as human MΦ reported a higher MHC-II expression in IM compared to AM [4,5,7,30]. Our own results resemble those findings, indicating that IM are more involved in acquired immune response than AM.

### Comparison to dendritic cells

Lung DC are a small subset of pulmonary mononuclear cells which exhibit low autofluorescence and are known to be loosely adherent [8,31-34]. Moreover, this cell type possesses an immature phenotype along with a rather weak CD83 expression [8]. Subsets of lung DC are known to express CD1a [31]. Both our MΦ preparations displayed high autofluorescence, were highly adherent and shared no phenotypic characteristics with DC regarding CD83 or CD1a expression. Therefore, the presence of DC in our cell preparations can be excluded for the most part.

### Phagocytic Activity

Both MΦ types displayed phagocytic activity, which underlines the macrophage phenotype of IM. Phagocytic activity was comparable in AM and IM. This finding resembles observations for Fcγ-dependent phagocytosis in the animal model [4]. Differences in phagocytic activity have been shown previously for human AM and IM phagocytosing *Saccharomyces cerevisiae*[6]. As this process is Fcγ-independent, these findings can not be compared to our results.

### Toll-like receptor expression

In the present study, expression of TLR1-10 mRNA levels were examined in both AM and IM for the first time. TLR1-10 mRNA expression by AM obtained from bronchoalveolar lavage was previously described by Maris et al. [35]. According to this study, TLR1, TLR4, TLR7, TLR8 are strongly and TLR2, TLR6 weakly expressed, whereas TLR3, TLR5, TLR9 and TLR10 were not detectable. Our results for TLR1, TLR4, TLR6, TLR8 and TLR10 expression by AM resemble those observations. In contrast to Maris et al., we observed a strong expression of TLR2 mRNA which is in line with results by Suzuki et al. [36] as well as ourselves [16]. Contrary to Maris et al. we were able to detect TLR3, TLR5 and TLR9 mRNA, suggesting a higher sensitivity of our assay. In fact, our real time PCR analysis is linear over 8 orders of magnitude, down to a concentration of 10^-6 ^attomole/μl.

It was long believed that both human monocytes as well as human macrophages do not express TLR9 [37]. This view was supported by examinations like that by Miettinen et al. [38], failing to detect TLR9 mRNA by Northern Blot in human macrophages. By now, the presence of TLR9 in MΦ was confirmed by Fenhalls et al. [39], Juarez et al. [40], and ourselves [16] by immunhistochemichal detection, Western blot analysis, flow cytometry, real time RT-PCR, and evidence of TLR9 functionality.

No significant differences between AM and IM concerning the TLR mRNA expression profile were found. Still, we can not exclude that TLR protein expression or localization differ in the different macrophage populations, which might both cause a differential cell reaction upon ligand binding.

### Cell reaction upon TLR activation

Previous studies using rat MΦ revealed that upon TLR4 stimulation AM express higher amounts of the proinflammatory cytokine TNF-α compared to IM [4,9]. Our data suggest that this is also true for human AM and IM. Moreover, we were able to detect significant differences between AM and IM concerning IL10 as well as IL6 expression.

Although IL10 is one of the most important anti-inflammatory mediators in human immune response [41,42], its expression by human AM and IM has not been investigated before. AM display low basal IL10 levels, which might allow efficient defence against inhaled particles and pathogens, whereas the fast induction of IL10 upon LPS treatment suggests an autoregulatory mechanism. As for IM, basal IL10 mRNA and protein levels were found to be significantly higher than in AM and to increase after LPS treatment. A recently published study comparing murine AM and IM showed that IL10 levels were markedly higher in IM [5]. Our data demonstrate that this is also true for human IM. In the animal model, IM were shown to inhibit lung DC maturation and migration in an IL10-dependent manner, thereby preventing Th2 sensitization to harmless inhaled antigens [5]. Our findings suggest that this might also be exhibited by human IM, indicating that IM play a crucial role in immune homeostasis.

IL6 has proinflammatory as well as anti-inflammatory properties. Studies using knockout mice demonstrated that in innate immunity IL6 acts predominantly as an antiinflammatory cytokine, mainly by attenuating the synthesis of proinflammatory cytokines [43,44]. Moreover, IL6 is involved in the specific immune response by upregulating B-cell differentiation, T-cell proliferation, and antibody secretion [44]. The high constitutive expression of IL6 that we found in IM both on mRNA and protein level indicates that IM display a pronounced immunoregulatory capacity and suggests that they are more involved in specific immune responses.

IL1ra is a major antiinflammatory cytokine that functions as a specific inhibitor of the two other functional members of the IL-1 family, IL-1a and IL-1β [41,45]. Our data demonstrate that IM secrete higher amounts of IL1ra when compared to AM, both at baseline and upon TLR4 activation. In contrast, LPS induced secretion of proinflammatory cytokines was low in IM when compared to AM (IL1β, IL12p40) or even completely absent (IL12p70 and IFNγ). These findings clearly underline the anti-inflammatory phenotype of IM previously described in the literature based on data obtained from murine or rat MΦ [4,5,9,24].

Despite the weak expression of TLR9 in AM and IM, cells reacted strongly upon stimulation with mycobacterial DNA. Methylation or digestion of mycobacterial DNA as well as chloroquine pretreatment lead to an abrogation of the macrophage response [16], which indicates that gene expression upon treatment with isolated DNA is not due to contaminants in DNA preparations, but due to TLR9 activation. It has been shown previously for human *in vitro *differentiated MΦ as well as a mouse MΦ cell line that TLR9 activation is higher upon treatment with bacterial DNA than after oligonucleotide treatment, which might be due to oligonucleotide structure [16,46,47]. Moreover, it has been reported for human *in vitro *differentiated macrophages as well as for AM from BAL that DNA from virulent strains has a lower potential to activate TLR9 in MΦ than DNA from attenuated strains [16]. Several studies indicate that the virulent H37Rv strain is able to methylate cytosines whereas the H37Ra strain is not [48,49], which might explain why H37Rv DNA fails to activate TLR9.

We were able to show that IM are less responsive to bacterial DNA than AM concerning TNF-α induction, which resembles our findings for TLR4 activation. Similar to the results of the TLR4 activation experiments, IL6 as well as IL10 expression were much higher in IM compared to AM, which clearly underlines the immunoregulatory function of IM.

Moreover, IL10 was only induced in IM, but not in AM, upon mycobacterial DNA treatment. Absence of IL10 induction after TLR9 activation by mycobacterial DNA has been reported before [40] and might be part of a mechanism of AM to overcome the immunosuppressive environment of the alveoli. Lung epithelial cells have been shown to constitutively express IL10, which is accompanied by an impaired responsiveness of AM towards IL10 [50]. In the same study, it was also observed that activation of human AM through TLR2, TLR4 or TLR9 leads to inhibition of IL10 receptor function associated with a reduced ability to activate STAT3. As IL10 is known to induce its own transcription *via *several positive feedback loops involving STAT3 [51,52], a low capacity of AM to activate STAT3 might contribute to the lack of IL10 induction in AM upon TLR9 activation as well as to the low basal levels of IL10 in comparison to IM.

### Conclusion

Taken together, the present results confirm and extend limited data obtained with murine and human AM and IM characterizing phenotypic differences. We were able to demonstrate functional and morphological differences as well as similarities between AM and IM from human lung tissue, leading to the conclusion that the heterogenity of lung macrophages should be taken into consideration in future studies on their role in TLR-mediated inflammatory response.

## Competing interests

The authors declare that they have no competing interests.

## Authors' contributions

JH, BD, TB, DM, LG, AM, CML, HH and AKK participated in design and coordination of the study.

Patients and samples were recruited by HH. JH carried out the flow cytometry and Real Time PCR assays. TB participated in flow cytometry assays. DM and JH measured cytokine protein profiles. Confocal microscopy was performed by RZ, electron microscopy by RZ and MK. JH wrote the manuscript to which AKK and BD added their contributions. AKK initiated and directed the study.

All authors read and approved the final manuscript.
